# Experimental Study on the Mechanical Properties of Diatomite-Modified Coastal Cement Soil

**DOI:** 10.3390/ma15217857

**Published:** 2022-11-07

**Authors:** Jiyuan Fang, Yunfeng Wang, Kefa Wang, Wenhao Dai, Yanfei Yu, Cuihong Li

**Affiliations:** 1 School of Civil Engineering, Shaoxing University, Shaoxing 312000, China; 2 Shaoxing Key Laboratory of Interaction between Soft Soil Foundation and Building Structure, Shaoxing 312000, China; 3 The Architecture Design & Research Institute of Zhejiang University Co., Ltd., Hangzhou 310027, China

**Keywords:** soil modification, coastal cement soil, diatomite, energy-dispersive spectroscopy, backscattered electron imaging

## Abstract

Diatomite is a non-metallic mineral resource rich in SiO_2_, which can be used to modify coastal cement soil. In order to explore the mechanical modification effect of diatomite on coastal cement soil at the age of 7 days, based on coastal cement soil with cement content of 5 % (mass fraction), diatomite of 0 %, 5 %, 10 %, 15 % and 20% (mass fraction) was mixed for modification. Through the unconfined compressive strength test, the triaxial unconsolidated undrained test, backscattered electron imaging (BSE), and energy-dispersive spectroscopy (EDS) technology, the influence of diatomite content and confining pressure on the peak strength of modified coastal cement soil was explored. The empirical formula between the peak strength of the DE specimen and the content of diatomite and confining pressure was established by curve fitting, and the fitting effect was ideal. When diatomite was mixed with coastal cement soil, the optimal dosage of diatomite was 5% from the perspective of mechanical properties and economic benefits of the maximum growth rate of compression and shear. The unconfined compressive strength test showed that the peak strength and elastic modulus of the modified coastal cement soil with 5% diatomite content were 37% and 57% higher than those of cement soil, respectively. The triaxial unconsolidated undrained test showed that the internal friction angle of the modified coastal cement soil was stable at about 30°, and cohesion of DE-5, DE-10, DE-15, and DE-20 increased by 28%, 48%, 78%, and 97%, respectively, compared to cement soil. The microscopic test found that the pore distribution of modified coastal cement soil is closely related to the strength change. The results show that the addition of diatomite can effectively improve the mechanical properties of soil-cement.

## 1. Introduction

Coastal soft soil is widely distributed in the world. Land subsidence caused by high water content, large pores, and low strength of soft soil hinders the development of coastal cities [1,2]. Uneven settlement caused by a soft soil foundation not only affects the quality of buildings but also brings about potential harm to later application. Direct curing of undisturbed soft soil can reduce the economic cost caused by excavation backfilling, so exploring the way of resource consumption is an effective measure to solve this problem [3].

Because cement can effectively improve soil properties in terms of strength, plasticity, and stiffness, many scholars have applied cement as a curing agent to improve soil that is not suitable for engineering construction. Ribeiro et al. [4] carried out unconfined compressive strength tests and microscopic analysis of cement soil and found that the hydration reaction of the active fine particles in cement can promote the production of more cementitious materials. These substances can agglomerate and fill the soil pores, which can promote the formation of a compact structure. Therefore, the compressive strength of soft soil can be improved. Mollamahmutoğlu et al. [5] found through triaxial tests that cement incorporation can effectively improve the shear performance of soft soil, and the improvement of shear performance mainly depends on the increase in the internal friction angle and cohesion. Walker [6] studied the compressive strength and bending strength of soil after cement stabilization and found that the anti-deformation ability of soft soil improves after cement incorporation. Cement plays a positive role in engineering seepage prevention and strength reinforcement, but cement production consumes a lot of energy and pollutes the atmosphere. The increase in carbon emissions exacerbates climate change and global warming, and dust pollution has a negative impact on human health [7,8,9]. In addition, soft soil after cement solidification usually is strongly alkaline, which easily pollutes groundwater and inhibits plant growth [10,11]. Therefore, it is important to find a modified material with good effects on and less pollution of soil.

To promote cement soil strength stability and alleviate environmental pollution, natural materials can be used to modify cement soil. Studies have found that diatomite can improve the mechanical properties of cement-based materials [12,13,14]. Diatomite is a biosilicite formed by ancient unicellular diatoms through long-term geological processes. It belongs to the non-metallic mineral resource family and is widely distributed around the world [15,16]. Diatomaceous earth has the characteristics of high sensitivity and strong structure. In places where diatomaceous earth accumulates, geological problems, such as landslides, instability, and foundation settlement, easily occur due to the influence of rainfall [17,18]. The excavation and use of diatomite are advantageous to reducing the safety risk caused by the large accumulation of diatomite, and it can modify coast soil. Mota et al. [19] studied the chemical composition of diatomite and found that the main component is SiO_2_, which has volcanic ash activity and is suitable for replacing some cementing materials. Moreover, the pH of diatomite is neutral, and the incorporation of soil has little effect on the pH of soil. Sardemir et al. [20] found that the incorporation of diatomite by mixing calcined diatomite into cement mortar could effectively improve the strength of mortar, and 15% was the optimal dosage. Rodriguez et al. [21] studied the compressive strength and resistance of diatomite-added concrete to chloride ion erosion. After the addition of diatomite, the porosity and pore size decreased, the strength increased by 15%, and the resistance to chloride ion erosion increased by 5% after 90 days. Cement is present in cement soil, concrete, and mortar, so there are many similarities in its reaction mechanism. For example, the material particles that need to be strengthened are cemented together under the influence of cement hydration, thereby enhancing the strength [22]. In view of this research, diatomite is mainly used in concrete and mortar modification, but there is no research on cement soil. Therefore, diatomite and cement soil are combined to improve the mechanical properties of cement soil and reduce carbon emissions and resource consumption.

In summary, in this paper, diatomite was used to modify coastal cement soil, and the influence of diatomite on the mechanical properties of coastal cement soil was discussed through the unconfined compressive strength test and the triaxial unconsolidated undrained test. The mechanism underlying diatomite-induced improvement of the mechanical properties of coastal cement soil was studied through backscattered electron imaging (BSE) and energy-dispersive spectroscopy (EDS) technology, which provided a reference for the modification application of coastal cement soil.

## 2. Materials, Experimental Scheme, and Process

### 2.1. Materials

**Figure 1:**
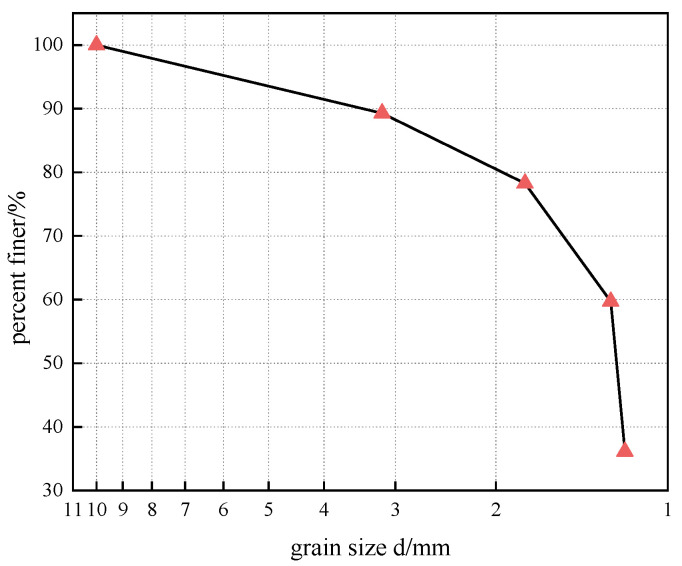
Grain-grading diagram of coastal soft soil with particle size below 2 mm.

The raw materials used in the experiment were cement, diatomaceous earth, and coastal soft soil. The soil used in the experiment was collected from the expansion project of the Shaoxing University of Arts and Sciences in Shaoxing City, Zhejiang Province. The region is located in the western part of Ningshao Plain, where marine sedimentary soft soil layers are widely distributed. The main physical parameters of the soil are shown in Table 1. According to the Test Methods of Soils for Highway Engineering (JTG 3430-2020), the soil classification was studied, which belongs to low-liquid-limit clay [23]. The soil with a particle size of less than 2 mm was selected as the test soil for particle gradation analysis, as shown in Figure 1. The P.O42.5 cement used in the test is produced by Shaoxing Zhaoshan Building Materials Co., Ltd. The main chemical composition is shown in Table 2, and the basic physical properties are shown in Table 3. Diatomite with a density of 0.47 g/cm^3^ was purchased from Linjiang Dayuan Diatomite New Materials Ecological Environmental Protection Technology Co., Ltd. (Linjiang, Jilin Province, China). The main chemical composition is shown in Table 2. The test water was tap water.

### 2.2. Design of the Test Plan

**Figure 2:**
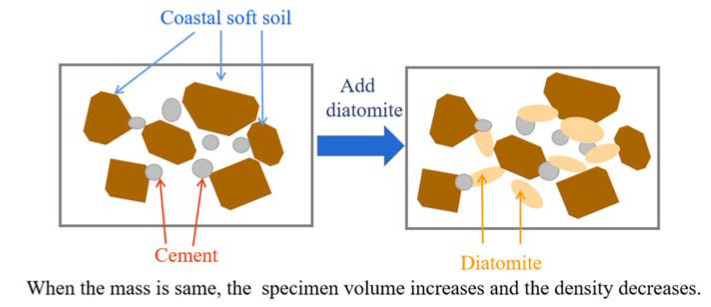
Schematic diagram of adding diatomite to cement soil.

To study the influence of different diatomite dosages on the mechanical properties of coastal cement soil, the diatomite dosages were preliminarily set at 0%, 5%, 10%, 15%, and 20%. According to the standard for the geotechnical testing method (GB/T50123-2019), the ring tool method was used to measure the sample density, as shown in Table 3 [24]. As shown in Table 4, the density decreased with the increase in diatomite content. This is mainly because with the same soil sample weight, diatomite particles covered the surface of cement soil particles and formed larger aggregates, which made the cement soil particles occupy more space, as shown in Figure 2. At the same time, diatomite has the characteristics of small density and large volume, thus reducing the density, so the diatomite incorporation amount should not exceed 20% [25].

Based on the preliminary test, the samples were finally divided into five groups, with five specimens in each group. The amounts of diatomite, cement, and water were determined according to Equations (1)–(3).

(1) $$C_{d}=M_{d}{/M}_{s}\times 100\%$$

(2) $$C_{c}=M_{c}{/M}_{s}\times 100\%$$

(3) $$C_{w}=M_{w}/(M_{s}+M_{c}+M_{d})\times 100\%$$

where *C_d_* is the diatomite content, *C_c_* is the cement content, and *C_w_* is the water content. The amounts of all these materials are mass fractions, and the unit is percentage (%). *M_d_* is the mass of diatomite, *M_c_* is the mass of cement, *M_s_* is the dry soil mass of soft coastal soil, *M_w_* is the mass of water, and the material unit is grams (g). The test scheme is shown in Table 5. In DE-*x,* DE represents diatomite-modified coastal cement soil and *x* represents the diatomite incorporation percentage. The determination of cement weight mainly depends on two aspects: On the one hand, the cement and soil content in similar projects in the Shaoxing area of Zhejiang Province is about 3–8% [26,27]; on the other hand, the cement content recommended by the cement-stabilizing material ratio test in the Technical Guidelines for Construction of Highway Roadbases (JTG/T F20-2015) is 5% [28].

### 2.3. Specimen Preparation

**Figure 3:**
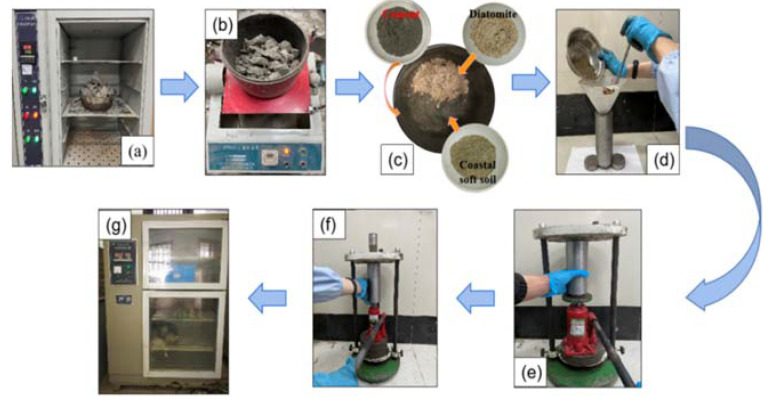
Standard specimen preparation diagram. (**a**–**g**). (**a**) is the drying soil in the process of sample preparation. (**b**) is the soil broken by the pulverizer during sample preparation. (**c**) is a mixture prepared during sample preparation. (**d**) shows the process of adding the mixture to the mold during sample preparation. (**e**) is a hydrostatic die during sample preparation to shape the soil sample. (**f**) shows the sample being pushed out of the die using jack pressure during the sample preparation process. (**g**) shows that the sample is prepared and put into the curing box for curing.

According to the Geotechnical Test Method Standard (GB/T 50123-2019) [24], the diameter and height of unconfined specimens and undrained triaxial specimens were 39.1 mm and 80.0 mm, respectively. The fabrication process is divided into the following steps:

(1) The coastal soft soil was baked in an oven at 105 °C for 24 h, as shown in Figure 3a. The dried coastal soft soil was then crushed by a crusher, as shown in Figure 3b. The crushed soft soil was passed through a 2-mm sieve, and particles below 2 mm were selected for further use.
(2) According to the experimental design requirements, the corresponding masses of cement and diatomite were weighed and added to the coastal soft soil with a particle size under 2 mm after screening, as shown in Figure 3c. After mixing evenly, the water mixer was added to stir for 5 min.
(3) A cushion block was put under the mold, filter paper was put on the cushion block, and a certain amount of mixture was taken with a funnel into the mold three times and evenly inserted, as shown in Figure 3d. The mixture was compacted each time after filling, and then the surface was scraped. After perfusion, filter paper was placed on the pad and finally put into the pad block.
(4) The mold filled with the mixture and the upper and lower cushion blocks were placed together on the upper part of the bearing plate of the 5t jack in the reaction frame, and the jack was shaken at a constant speed until the upper and lower cushion blocks were pressed into the mold, as shown in Figure 3e. The static pressure of the jack was maintained for about 1 min, and then the pressure was lifted. Finally, the cylinder specimen with a diameter of 39.1 mm and a height of 80 mm was taken out, as shown in Figure 3f. Next, a plastic film was sealed on the surface of the specimen.
(5) After specimen preparation, the specimen was put into a standard curing box for 7 days, as shown in Figure 3g. The curing temperature was controlled at 20 ± 2 °C, and the relative humidity was >95%.

### 2.4. Test Method

#### 2.4.1. Unconfined Compressive Strength Test

The unconfined compressive strength (UCS) test equipment included the TKA-WCY-1F automatic unconfined compressive test system produced by Nanjing Techao Technology Co., Ltd., (Nanjing, China), and the test shear speed was set to 1 mm/min. The maximum axial load the UCS tester can withstand is 10 KN, the instrument accuracy is 1 N, and the sensor accuracy is 0.1%. The tests were stopped when the strain reached 8%. Each test was repeated five times, and the average values were used for subsequent analyses.

#### 2.4.2. Triaxial Unconsolidated Undrained Test

The instrument used in the triaxial unconsolidated undrained (UU) test was the TKA-TTS-3S automatic triaxial apparatus produced by Nanjing Tekao Technology Co., Ltd. The coastal cement soil specimens with different proportions of diatomite were tested under confining pressures of 100 kPa, 200 kPa, 300 kPa, and 400 kPa. The maximum axial load that the UU tester can withstand is 10 KN, the instrument accuracy is 0.1 N, and the sensor accuracy is 0.1%. The tests were terminated when the stress–strain curve stabilized or the axial strain reached 15%.

#### 2.4.3. Microscopic Tests

BSE and EDS were performed, as per previous research by Uzarowicz [29]. The BSE test instrument included an SU3800 scanning electron microscope, which is produced by Hitachi Corporation. First, the dried specimens were sprayed with gold, and then the specimens were put into the test bench to take pictures. The elements were analyzed using an E2359 spectrometer produced by EDAX (Mahwah, NJ, USA).

## 3. Results and Discussion

### 3.1. Unconfined Compressive Strength Test

#### 3.1.1. Peak Strength Analysis

**Figure 4:**
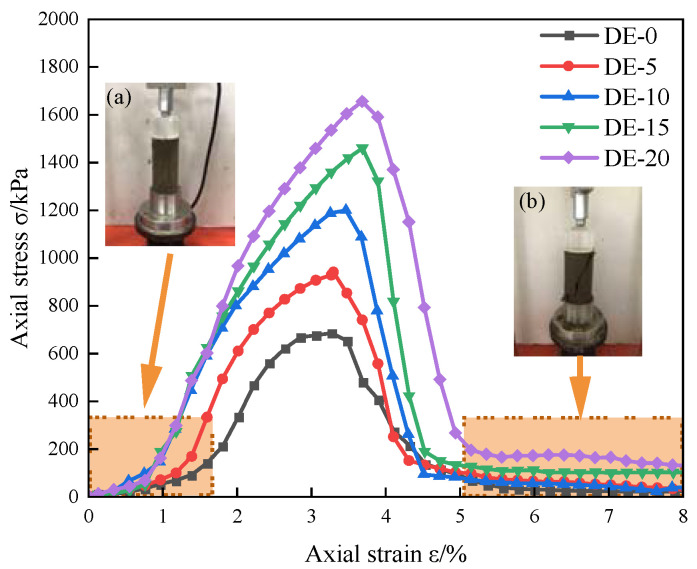
Stress–strain curves of coastal cement soil modified with different amounts of diatomite. (**a**) and (**b**) are the initial and failure images of specimens, respectively.

The results obtained by testing only one sample were not representative and reproducible because of the contingency of the test. Testing errors can be reduced by multiple tests on samples, so five parallel tests were carried out on the same group of samples in this paper. At the same time, to reduce the data discretization caused by test errors, the weighted average was used to obtain the normalized curve of the five groups of test data after normalization [30]. Figure 4 is the stress–strain curve of coastal cement soil modified with different amounts of diatomite. As shown in Figure 4, the stress–strain curves of coastal cement soil with different diatomite contents are of the softening type, and the unconfined compressive strength increased with the increase in diatomite content, which is consistent with the experimental results of Fenglei et al. [31]. Figure 4 shows that DE-5, DE-10, DE-15, and DE-20 curves were 37%, 75%, 114%, and 142% higher than that of DE-0. From the perspective of growth rate, the peak strength of DE-5, DE-10, DE-15, and DE-20 increased by 37%, 28%, 22%, and 13%, respectively, compared to the previous dosage, and the fastest growth rate of peak strength was observed with DE-5. At the same time, according to the Technical Regulations for Construction of Highway Pavement Base (JTG/T F20-2015)[28], when the content of diatomite reaches 10%, it can meet the strength requirements of the base of secondary and below medium and light traffic highway. Therefore, this study has certain engineering significance.

The increase in the strength of coastal cement soil mixed with diatomite is mainly due to the large amount of active SiO_2_ in diatomite. This active SiO_2_ can react with the cement hydration product Ca(OH)_2_ to generate hydrated calcium silicate gel (C-S-H), and C-S-H has strong cementation, thus improving the compressive strength of coastal cement soil [32]. With the increase in diatomite content, the peak strength still increased. This may be because with the increase in diatomite content, the excitation effect of cement is limited, and diatomite without a hydration reaction gradually increases. These particles can fill the soil pores, improve the density of the specimen, and thus improve the strength of the specimen [33].

#### 3.1.2. Elastic Modulus Analysis

The elastic modulus is an index to measure the difficulty in the elastic deformation of soil [34]. The larger the value, the smaller the possibility of soil deformation under the same deformation conditions. The calculation formula is as follows [35]:

(4) $$E_{50}=\frac{\Delta F}{\Delta \varepsilon }$$

**Figure 5:**
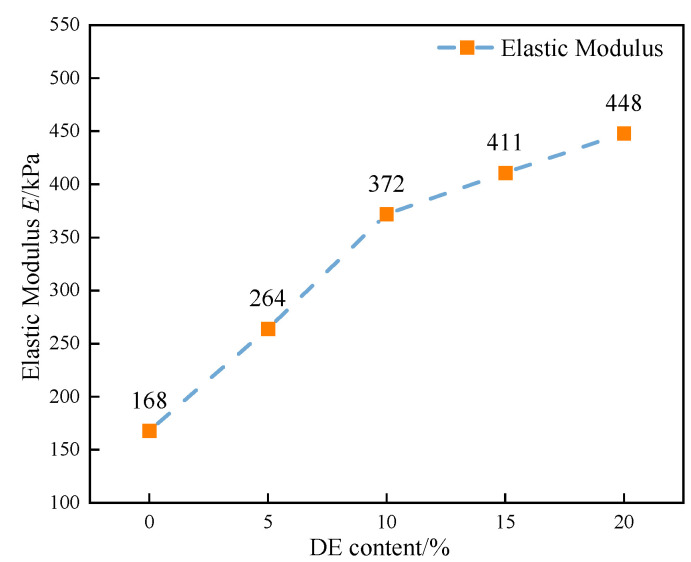
The elastic modulus of coastal cement soil specimens modified with different amounts of diatomite.

In Equation (4), ΔF is half of the peak strength and Δε is the corresponding strain when the strength is ΔF. Figure 5 shows the elastic modulus of coastal marine soil specimens modified with different dosages of diatomite. According to Figure 5, the elastic modulus of DE-5 was 1.57 times that of DE-0, the elastic modulus of DE-10 was 1.41 times that of DE-5, and the elastic modulus of DE-15 was 1.10 times that of DE-10. The elastic modulus of DE-20 was 1.09 times that of DE-15. The peak strength of the specimen increased continuously, but the corresponding strain changed little. Therefore, the elastic modulus of the specimen increased continuously with the increase in diatomite content. The growth rate first increased and then decreased, which is similar to the growth law of the peak strength of unconfined compressive strength.

### 3.2. Unconsolidated Undrained Triaxial Test

#### 3.2.1. Stress–Strain Curve

**Figure 6:**
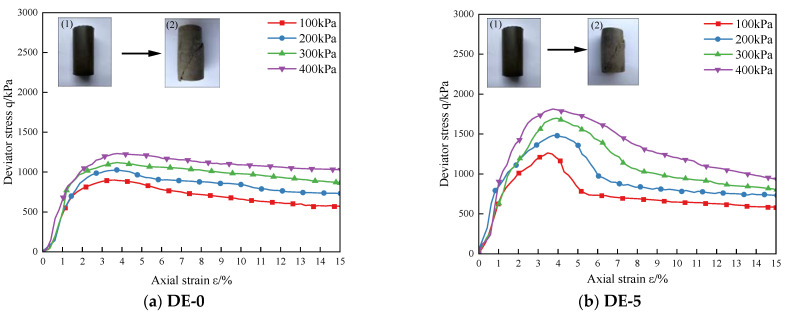

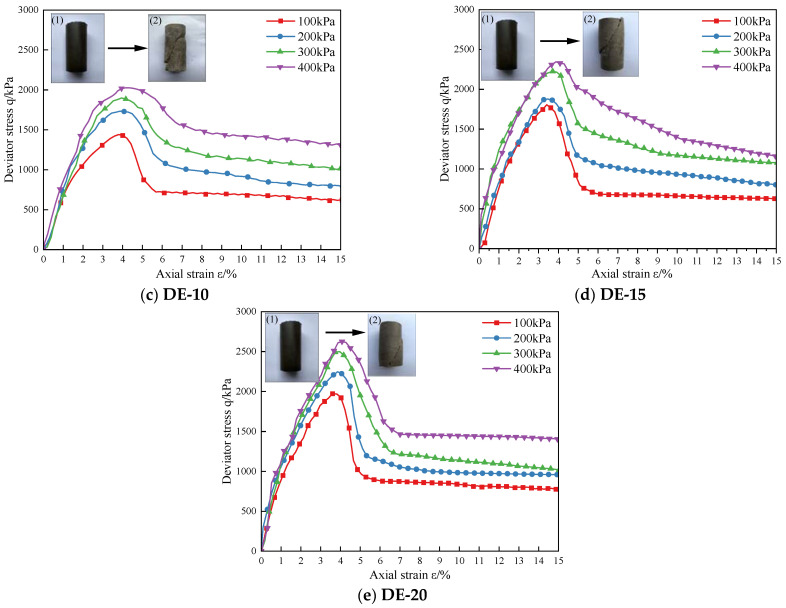
Deviatoric stress–strain relationship curves of specimens with different diatomite contents. (1) and (2) are the initial and failure images of specimens, respectively.

Figure 6 shows the deviatoric stress q versus axial strain for the DE specimens. Observing Figure 6, with the addition of diatomite, the principal stress difference gradually increased and the curve showed a downward trend after peaking, and with the increase in diatomite content, the softening trend became gradually obvious. In Figure 6, (1) and (2) are the initial and failure images of specimens, respectively. Observing the failure modes, it can be found that the failure modes of the specimens are all shear failures. Under the action of confining pressure, the particle–pore–water interaction in the soil structure produces shear stress. With the decrease in the effective stress, the soil skeleton cannot bear the external pressure eventually, a weak surface gradually forms, and then shear failure occurs [36].

#### 3.2.2. Strength Curves and Shear Strength Parameters

**Figure 7:**
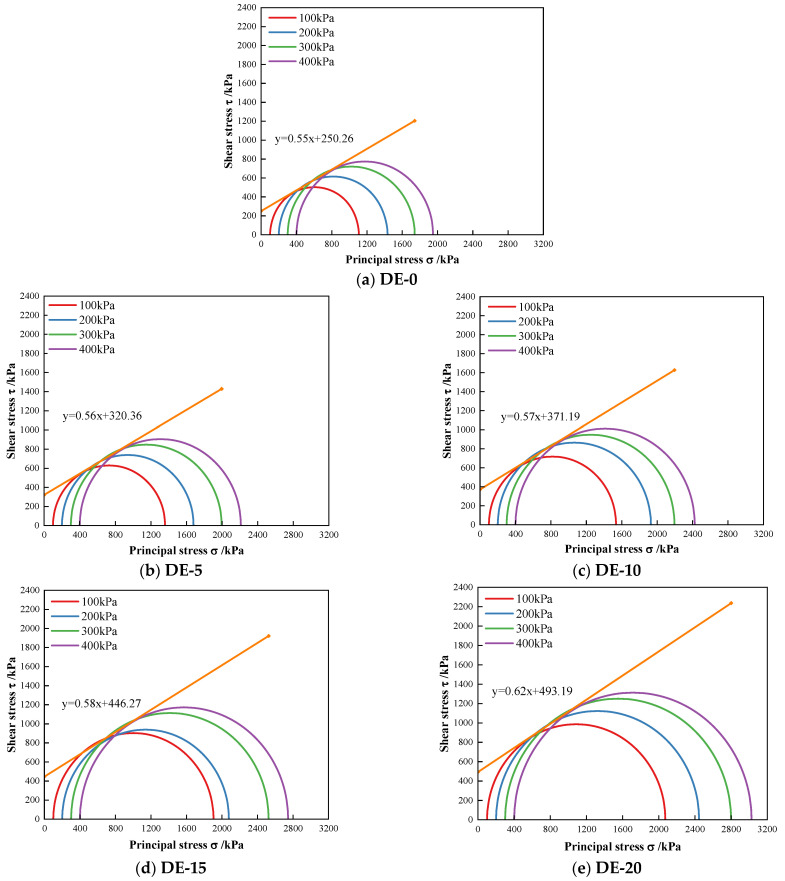
Mohr envelope of specimens with different diatomite contents.

Taking the peak value of the deviatoric stress as the failure point, the normal stress σ as the abscissa, and the shear stress τ as the ordinate, the strength envelope of the specimen can be drawn [37]. On the abscissa, with $\frac{\sigma_{1}+\sigma_{3}}{2}$ as the center and $\frac{\sigma_{1}-\sigma_{3}}{2}$ as the radius, the ultimate stress Mohr circle with different diatomite contents was drawn on the τ–σ stress plane diagram, as shown in Figure 7. Next, the common tangents of the Mohr circle under four different confining pressures were drawn, that is, the shear strength envelope of the Mohr circle under ultimate stress, in which σ*_1_* represents the major principal stress and σ*_3_* represents the minor principal stress.

From the envelope of the shear strength, the shear strength parameters c and φ and the shear strength formula of the specimens with different diatomite contents can be obtained, as shown in Table 5. Observing the c and φ data of different diatomite contents in Table 6, it can be found that with the increase in diatomite contents, the internal friction angle of the specimen increased continuously. After adding diatomite, the internal friction angle of the specimen improved to a certain extent, but it was basically stable at about 30°. The cohesion increased significantly with the change in diatomite content, which indicated that the cohesion is more sensitive to the change in the shear strength of DE specimens. Compared to DE-0, the cohesion of DE-5, DE-10, DE-15, and DE-20 increased by 28%, 48%, 78%, and 97%, respectively. From the perspective of each increase, the increase was 28%, 16%, 20%, and 11%, and the highest increase was with DE-5. The increase in the internal friction angle and cohesion is mainly due to a series of chemical reactions taking place inside the soil specimen, resulting in cementation and promoting the connection between soil particles [38]. When the content of diatomite is more than 5%, the increase in cohesion is relatively low because the cement and soil, cement and diatomite, and diatomite and soil in the specimen fully react. The continuous increase in the content mainly plays the role of filling the pores [39].

#### 3.2.3. Peak Intensity Empirical Formula

**Figure 8:**
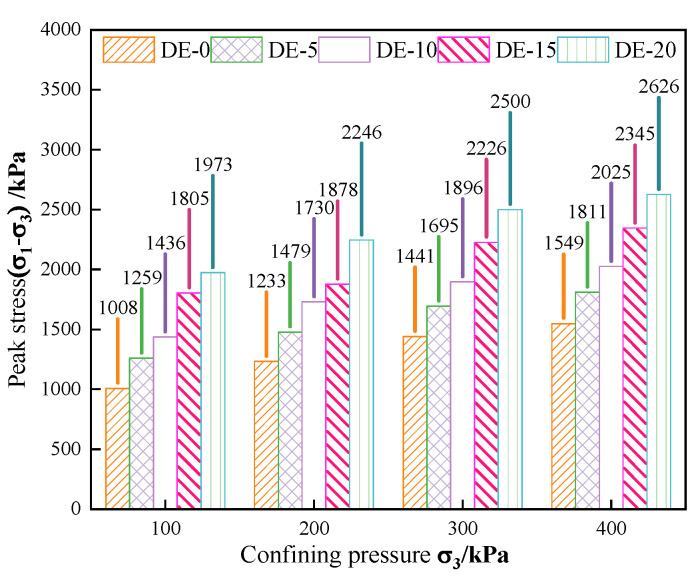
Peak intensity of specimens with different diatomite contents under different confining pressures.

Analysis of the peak strength of the specimen can help to gain a better understanding of the shear failure resistance of the specimen. Figure 8 shows the peak intensities of specimens with different diatomite contents under confining pressures of 100 kPa, 200 kPa, 300 kPa, and 400 kPa.

It can be seen from Figure 8 that when the content of diatomite remained unchanged, with the increase in confining pressure, the peak intensity of DE specimens increased continuously. Under the same confining pressure, with the increase in diatomite content, the peak strength of DE specimens also increased continuously. From the peak intensity, the maximum peak intensity occurred with DE-20. When the confining pressures were 100 kPa, 200 kPa, 300 kPa, and 400 kPa, the peak strength of DE-20 increased by 96%, 82%, 73%, and 70%, respectively, compared to DE-0. From a growth rate perspective, the largest increase in peak intensity occurred with DE-5. The improvement of DE-5 over DE-0 was 25%, 20%, 18%, and 17%, respectively, for confining pressures of 100 kPa, 200 kPa, 300 kPa, and 400 kPa.

In conclusion, there is a close relationship between the peak intensity and the content of diatomite and confining pressure. To further explore the relationship between the peak strength (PS) of diatomite-modified coastal marine soil and the diatomite content (D) and confining pressure (CP), a binary first-order polynomial was used to determine the relationship between DE-0 and DE-5. The measured data of DE-10 and DE-15 under 100 kPa, 200 kPa, 300 kPa, and 400 kPa confining pressures were fitted, and the empirical formula of the following Equation (5) was obtained:

PS = 580 + 61 × D + 2 × CP(5)

**Figure 9:**
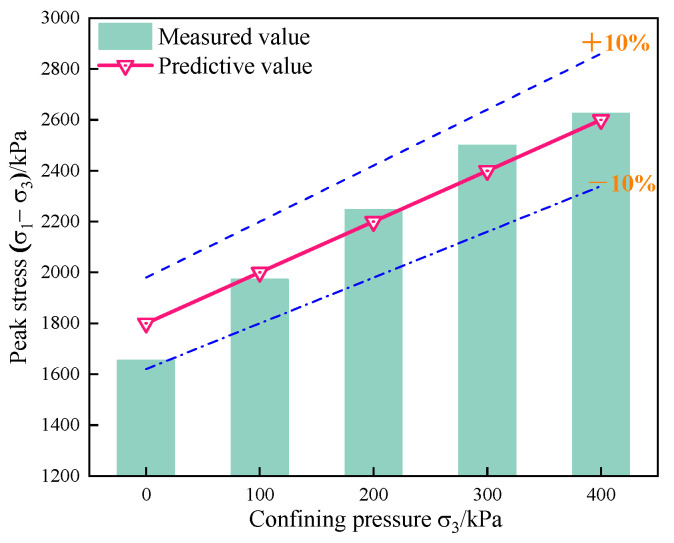
Comparison of the measured and predicted peak intensities with 20% diatomite content.

The fitting accuracy (R^2^) was 0.96, indicating that the model has a good degree of fit. The measured diatomite content and confining pressure were substituted into the empirical formula, and the fitted value was compared with the measured value. The measured value was both above and below the predicted value. This empirical formula was used to predict the peak strength of the specimen under different confining pressures when the diatomite content was 20%, as shown in Figure 9. According to Figure 9, it can be seen that although the empirical formula had errors, the overall error was controlled within 10%. Comparing the predicted value with the measured value, it was found that under confining pressures of 0 kPa, 100 kPa, 200 kPa, 300 kPa, and 400 kPa, the absolute value of the error between the predicted value and the measured value was 9%, 1%, 2%, 4%, and 1%, respectively. Although the error size was different, they were all less than 10%. This shows that the established empirical formula has good predictive ability and can be used to predict the peak strength of coastal seawater soil mixed with different diatomite contents and under different confining pressures at 7 days of age, which has certain practical engineering significance.

### 3.3. Microscopic Analysis

**Figure 10:**
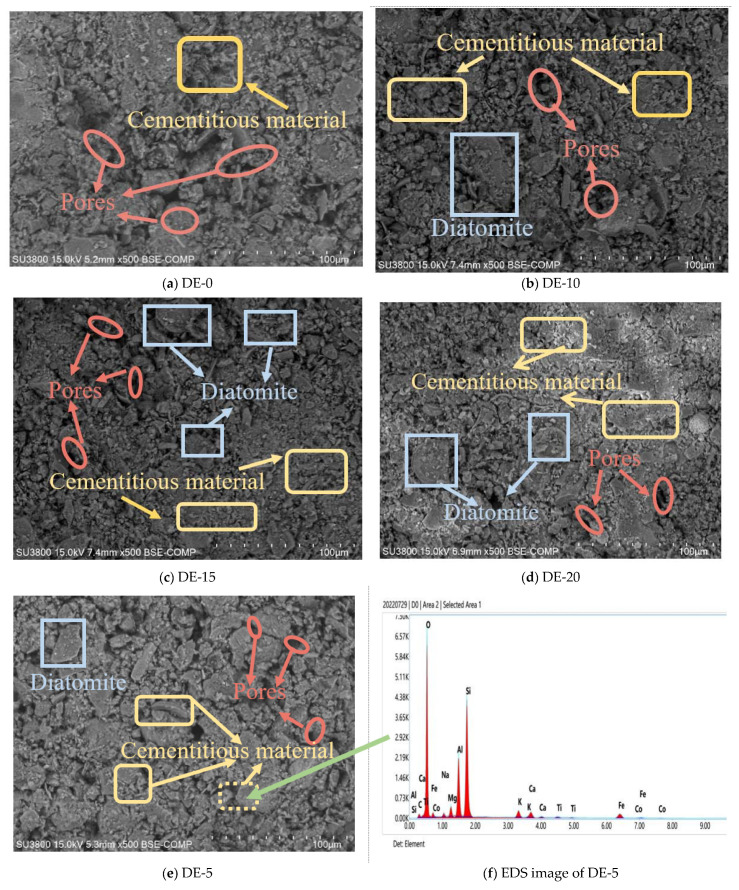
BSE photos and EDS images of coastal marine soil specimens modified with different amounts of diatomite. (**a**) BSE image of DE-0,500 times, (**b**) BSE image of DE-10,500 times, (**c**) BSE image of DE-15,500 times, (**d**) BSE image of DE-20,500 times, (**e**) BSE image of DE-0,500 times, and (**f**) EDS image of DE-5.

To understand the effect of diatomite modification on coastal marine soil, BSE tests were carried out on coastal marine soils with different dosages of diatomite, and the test results are shown in Figure 10. From Figure 10a, it can be observed that the sample soil particles are wrapped by cement hydrate to form large particles, but the degree of cementation among particles is poor, and the overall skeleton is dispersed. From Figure 10a–e, it can be clearly seen that the number of agglomerates continued to increase after the addition of diatomite, on the one hand. On the other hand, with the increase in the amount of diatomite, the size of the residual diatomite decreased first and then increased later. The gradually decreasing size of diatomite in the BSE photo is due to the reaction between amorphous SiO_2_ in diatomite and Ca(OH)_2_ produced by cement hydration, resulting in flocculent and flake-like cementitious materials. The energy spectrum test of the cemented part in the microstructure of DE-5 showed that the flocculent product was C-S-H, as shown in Figure 10f. These gels can cohere loose soil particles into large flaky aggregates, enhancing the integrity and compactness of coastal marine soils [40]. With the increase in diatomite content, the residual diatomite particles in the BSE image gradually increased and became larger. This may be due to the increase in diatomite content, the amount of cement being less than that of diatomite, and the hydration reaction activity being relatively low. Additionally, in cement soil, due to the existence of soil particles, cement and soil particles may adhere together and some of the diatomite incorporated cannot fully react, and this diatomite plays a greater role in filling pores in soil [41].

**Figure 11:**
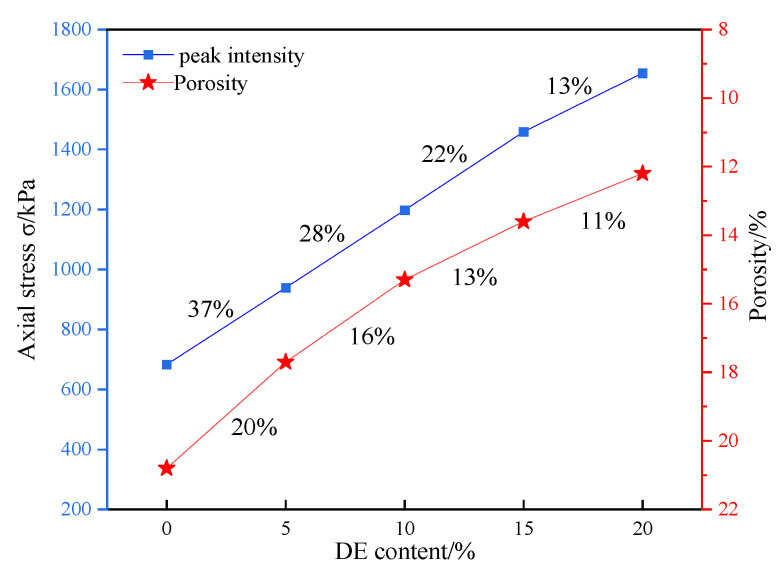
Relationship between the porosity and strength of diatomite-modified coastal cement soil; the intensity or porosity of diatomite varied from one dosage to the next.

The microscopic pore structure in the BSE images of coastal marine soil with different amounts of diatomite was analyzed by the image processing software Image-Pro Plus. The image is binarized and thresholded, the gray value of the image is adjusted to separate the particles in the image from the background, and the image is converted into a black image, where the black areas represent holes [42]. By calculating the porosity, the soft soil structure can be quantitatively analyzed. The porosity is shown in Figure 11. It can be seen from Figure 11 that with the increase in the content of diatomite, the porosity of the specimens decreased continuously. The maximum amplitude appeared when the content of diatomite was 5%, which is consistent with the increasing law of unconfined compressive strength.

## 4. Conclusions

Modification of coastal soft soil with diatomite and cement can improve its mechanical properties so that the modified coastal soft soil can be used in road engineering and realize the effective use of coastal soft soil. In this paper, the effects of diatomite on the strength, deformation, and shear properties of coastal marine soil were studied by the unconfined compressive strength test and the triaxial unconsolidated undrained test. In addition, the microscopic mechanism was analyzed using BSE and EDS. The main conclusions are as follows:

(1) The stress–strain curve of diatomite mixed with coastal seawater is softened. This softening trend is more pronounced in the triaxial unconsolidated undrained test than in the unconfined compressive strength test. With the same content of diatomite, the peak strength increases continuously with the increase in confining pressure.
(2) The peak stress of coastal marine soil increases with the increase in diatomite content. When the content of diatomite is 5%, the amount of its strength increases most under different confining pressures. Under confining pressures of 0 kPa, 100 kPa, 200 kPa, 300 kPa, and 400 kPa, the strength of DE-5 increased by 37%, 25%, 20%, 18%, and 17%, respectively, compared to DE-0. An empirical formula of peak strength related to confining pressure and diatomite content was established, and the error of the formula was controlled within 10%, which has certain practical significance in engineering.
(3) With the addition of diatomite, the elastic modulus, internal friction angle, and cohesion of the specimens were continuously enhanced. Unconfined compressive strength tests yielded at least a 9% increase in the elastic modulus. In addition, the growth law of elastic modulus is similar to that of compressive strength. From the triaxial unconsolidated undrained test, the internal friction angle was basically stable at about 30° after the addition of 5%, 10%, 15%, and 20% diatomite. The cohesion was 28%, 48%, 78%, and 97% higher, respectively, than that of cement soil, respectively.
(4) The incorporation of diatomite produces a large number of flocculent and flake-like cementitious materials inside the soil, which are the product of a pozzolanic reaction and can play a role in curing coastal soft soil. In addition, the quantitative analysis of the microscopic pore area shows that with the increase in diatomite content, the porosity decreases continuously, and the maximum decrease in porosity is 20%. The results of the ultimate compressive strength test are basically the same.

In this paper, the mechanical properties of coastal cement soil with diatomite at 7 days of age were studied, and the effect of diatomite at 28 days of age on the mechanical properties of coastal cement soil still needs to be further studied. The mechanical tests mainly include the unconfined compression test and the triaxial unconsolidated undrainage test, which can be supplemented by the California load-bearing ratio test and the dynamic triaxial test to further analyze the changes in the mechanical properties of coastal cement soil after diatomite modification. At the same time, the proctor method can be improved for subsequent testing to further understand how the presence of diatoms affects the optimal maximum density–moisture content relationship.

## Figures and Tables

**Table 1:** Physical index of soil.

Indicator Name	Natural Moisture Content (%)	Plastic Limit (%)	Liquid Limit (%)	Plasticity Index	Liquid Index	Maximum Dry Density (g/cm^3^)
Numerical value	35	18.43	44.74	26.31	0.63	1.65

**Table 2:** Main chemical composition of diatomite and P.O42.5 cement (mass fraction).

Chemical Composition	SiO_2_	Fe_2_O_3_	Al_2_O_3_	CaO	MgO
Cement	19.87	3.40	4.90	65.10	1.25
Diatomite	86.80	1.25	2.05	0.45	0.30

**Table 3:** Basic physical and mechanical indexes of P.O42.5 cement.

Fineness (%)	Initial Setting Time (min)	Final Setting Time (min)	3-Day Compressive Strength (MPa)	28-Day Compressive Strength (MPa)	3-Day Flexural Strength (MPa)	28-Day Flexural Strength (MPa)
3.4	210	295	29.6	48.1	6.0	9.0

**Table 4:** Specimen density table.

Specimen	DE-0	DE-5	DE-10	DE-15	DE-20
Density (g/cm^3^)	2.13	1.93	1.71	1.57	1.47

**Table 5:** Experimental design scheme.

Specimen	Moisture Content (%)	Diatomite Dosage (%)	Cement Dosage (%)	Conservation Age (days)
DE-0	25	0	5	7
DE-5		5	5	
DE-10		10	5	
DE-15		15	5	
DE-20		20	5	

**Table 6:** Shear strength parameters of coastal cement soil with different diatomite contents.

Diatomite Dosage (%)	Intensity Envelope	Internal Friction Angle *φ* (°)	Cohesion *c* (kPa)
DE-0	τ = 0.55x + 250.26	28.73	250.26
DE-5	τ = 0.56x + 320.36	29.05	320.36
DE-10	τ = 0.57x + 371.19	29.75	371.19
DE-15	τ = 0.58x + 446.27	30.28	446.27
DE-20	τ = 0.62x + 493.19	31.90	493.19

## Data Availability

Not applicable.
